# Crystal structure of (2*S*,4*S*)-5,5-dimethyl-2-(pyridin-2-yl)-1,3-thia­zolidine-4-carb­oxy­lic acid

**DOI:** 10.1107/S1600536814024854

**Published:** 2014-11-15

**Authors:** Payel Laskar, Naoto Kuwamura, Nobuto Yoshinari, Takumi Konno

**Affiliations:** a Department of Chemistry, Graduate School of Science, Osaka University, Toyonaka, Osaka 560-0043, Japan; b CREST, Japan Science and Technology Agency, Toyonaka, Osaka 560-0043, Japan

**Keywords:** crystal structure, thia­zolidine, hydrogen bonding, C—H⋯π contacts

## Abstract

In the title compound, C_11_H_14_N_2_O_2_S, the thia­zolidine ring has an envelope conformation with the C atom bonded to the carb­oxy­lic acid group at the flap. Two C atoms of the thia­zolidine ring adopt *S* conformations. In the crystal, O—H⋯N hydrogen bonds between the amine and carb­oxy­lic acid groups construct a helical chain structure along the *a-*axis direction. The chains are further connected *via* weak C—H⋯π contacts, forming a layer parallel to the *ac* plane.

## Related literature   

For background to compounds containing thia­zoline or thia­zolidine rings, see: Bolos *et al.* (2002 ▶); Pontiki *et al.* (2006 ▶); Shih & Ke (2004 ▶). For related structures, see: Brunner *et al.* (1984 ▶, 2001 ▶). For the preparation of d-penicillamine-coordinated metal complexes, see: Igashira-Kamiyama & Konno (2011 ▶).
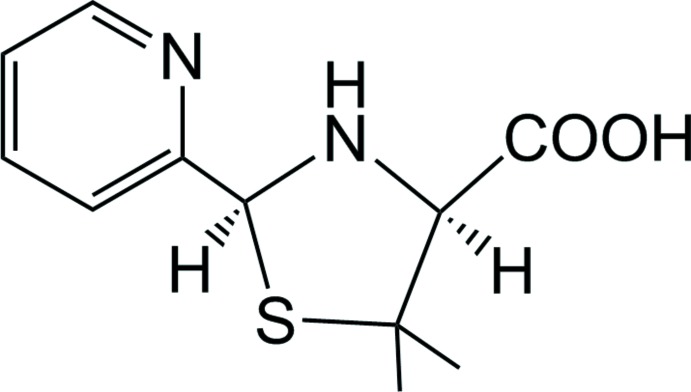


## Experimental   

### Crystal data  

C_11_H_14_N_2_O_2_S
*M* *_r_* = 238.30
Orthorhombic, 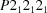

*a* = 7.906 (4) Å
*b* = 11.306 (5) Å
*c* = 13.504 (7) Å
*V* = 1207.1 (10) Å^3^
*Z* = 4
Mo *K*α radiation
μ = 0.26 mm^−1^
*T* = 200 K
0.25 × 0.25 × 0.25 mm

### Data collection  

Rigaku R-AXIS RAPID diffractometer
Absorption correction: multi-scan (*ABSCOR*; Higashi, 1995 ▶) *T*_min_ = 0.785, *T*_max_ = 0.938
9629 measured reflections
2767 independent reflections
2711 reflections with *F* ^2^ > 2σ(*F* ^2^)
*R*_int_ = 0.020

### Refinement  

*R*[*F* ^2^ > 2σ(*F* ^2^)] = 0.028
*wR*(*F* ^2^) = 0.074
*S* = 1.10
2767 reflections
152 parameters
H atoms treated by a mixture of independent and constrained refinement
Δρ_max_ = 0.23 e Å^−3^
Δρ_min_ = −0.18 e Å^−3^
Absolute structure: Flack *x* determined using 1118 quotients [(*I* ^+^)−(*I* ^−^)]/[(*I* ^+^)+(*I* ^−^)] (Parsons *et al.*, 2013 ▶)
Absolute structure parameter: 0.01 (9)

### 

Data collection: *PROCESS-AUTO* (Rigaku, 1998 ▶); cell refinement: *PROCESS-AUTO*; data reduction: *PROCESS-AUTO*; program(s) used to solve structure: *SIR92* (Altomare *et al.*, 1993 ▶); program(s) used to refine structure: *SHELXL2013* (Sheldrick, 2008 ▶); molecular graphics: *CrystalStructure* (Rigaku, 2014 ▶); software used to prepare material for publication: *CrystalStructure*.

## Supplementary Material

Crystal structure: contains datablock(s) New_Global_Publ_Block, I. DOI: 10.1107/S1600536814024854/is5377sup1.cif

Structure factors: contains datablock(s) I. DOI: 10.1107/S1600536814024854/is5377Isup2.hkl

Supporting information file. DOI: 10.1107/S1600536814024854/is5377Isup3.cml

**Fig. 1.**
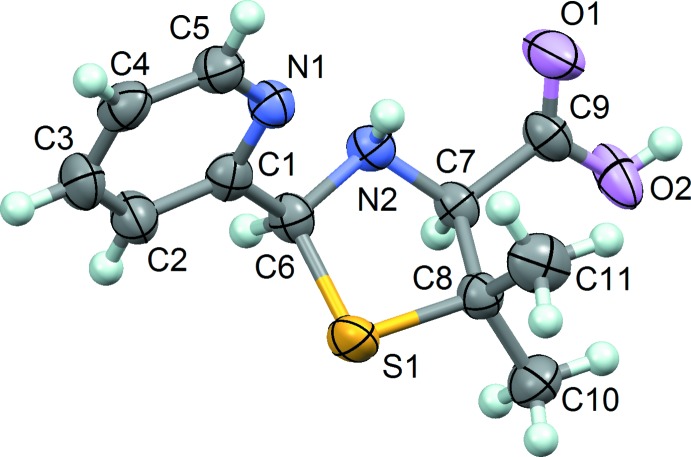
Molecular structure of the title compound with the atom numbering scheme. Displacement ellipsoids are at the 70% probability level. H atoms are drawn as spheres of arbitrary radii.

**Fig. 2.**
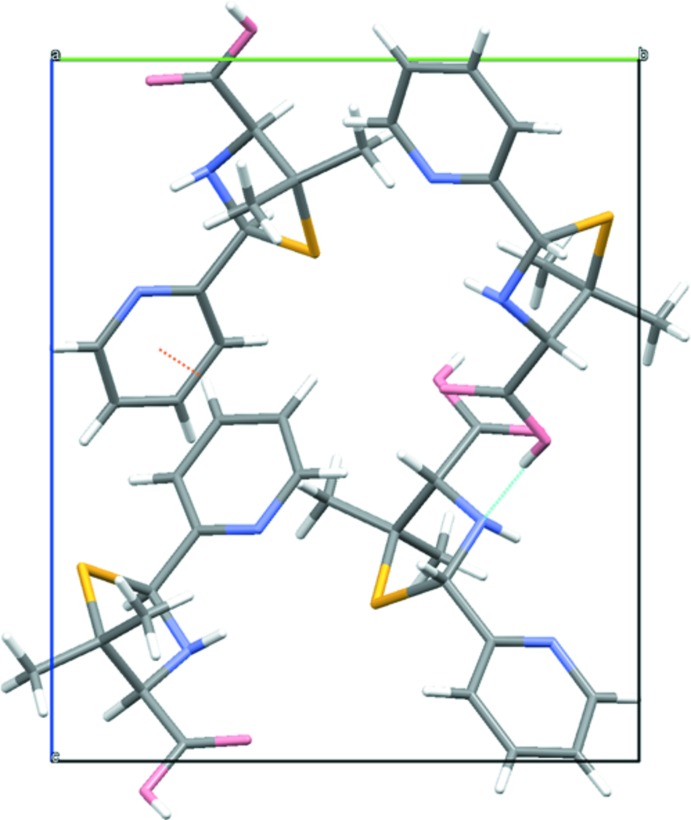
Crystal packing diagram of the title compound, viewed along with the a axis. Orange and blue dotted lines indicate the weak C—H···π contact and the O—H···N hydrogen bond, respectively.

. DOI: 10.1107/S1600536814024854/is5377fig1.tif
Mol­ecular structure of the title compound with the atom numbering scheme. Displacement ellipsoids are at the 70% probability level. H atoms are drawn as spheres of arbitrary radii.

a . DOI: 10.1107/S1600536814024854/is5377fig2.tif
Crystal packing diagram of the title compound, viewed along with the *a* axis. Orange and blue dotted lines indicate the weak C—H⋯π contact and the O—H⋯N hydrogen bond, respectively.

CCDC reference: 1033831

Additional supporting information:  crystallographic information; 3D view; checkCIF report

## Figures and Tables

**Table 1:** Hydrogen-bond geometry (, ) *Cg* is the centroid of the N1/C1C5 ring.

*D*H*A*	*D*H	H*A*	*D* *A*	*D*H*A*
O2H13N2^i^	0.79(3)	1.87(3)	2.654(2)	173(3)
C3H3*Cg* ^ii^	0.95	2.81	3.629(2)	145

Symmetry codes: (i) 
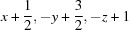
; (ii) 
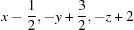
.

## supplementary crystallographic information

### S1. Structural commentary

The compounds containing thia­zoline or thia­zolidine rings are of attractive attention for their coordination chemistry and potential anti­biotic and anti­tumoral activities (Pontiki *et al.*, 2006; Shih & Ke, 2004; Bolos *et al.*, 2002). As part of our continuing study to create sulfur coordinated coordination compounds (Igashira-Kamiyama & Konno, 2011), we synthesized a novel thia­zolidine compound, which is prepared from the condensation of *D*-penicillamine and 2-pyridine carboxaldehyde. Herein the structure and synthesis of (2*S*,4*S*)-5,5-di­methyl-2-(pyridin-2-yl)thia­zolidine-4-carb­oxy­lic acid are reported.

The title compound is enanti­ometrically pure and the absolute structure was determined by the refinement of the Flack parameter [0.01 (9)]. The chiral C-2 and C-4 atoms (atoms C6 and C7, respectively) have *S* configurations (Fig. 1). In the crystal, the molecules are inter­acted through O—H···N hydrogen bonds and weak C–H···π contacts (Table 1), forming a layer parallel to the *ac* plane (Fig. 2).

### S2. Synthesis and crystallization

To a white suspension of *D*-penicillamine (60 mg, 0.40 mmol) in MeOH (2.5 mL) was added 2-pyridine carboxaldehyde (43 mg, 0.40 mmol). The mixture was stirred at 50 °C for 2 h to give a pale yellow solution. The reaction mixture was allowed to stand at room temperature. Colorless crystals were obtained by slow evaporation of the reaction mixture after 10 days. Yield: 38 mg (40%). Anal Calcd for C_11_H_14_N_2_O_2_S: C 55.44, H 5.92, N 11.71%. Found: C 55.20, H 5.82, N 11.71%. IR: *ν*_max_ (cm^-1^): 1570, 1591.

### S3. Refinement

C-bound H atoms were placed at calculated positions (C—H = 0.95, 0.98, or 1.00 Å) and refined as riding, with *U*_iso_(H) = 1.2*U*_eq_(C). N and O-bound H atoms were located in a difference Fourier map and their positions were refined with *U*_iso_(H) = 1.5*U*_eq_(N or O).

### Crystal data

**Table:**

C_11_H_14_N_2_O_2_S	*D*_x_ = 1.311 Mg m^−^^3^
*M**_r_* = 238.30	Mo *K*α radiation, λ = 0.71075 Å
Orthorhombic, *P*2_1_2_1_2_1_	Cell parameters from 606 reflections
*a* = 7.906 (4) Å	θ = 3.0–21.8°
*b* = 11.306 (5) Å	µ = 0.26 mm^−^^1^
*c* = 13.504 (7) Å	*T* = 200 K
*V* = 1207.1 (10) Å^3^	Block, colorless
*Z* = 4	0.25 × 0.25 × 0.25 mm
*F*(000) = 504.00	

### Data collection

**Table:**

Rigaku R-AXIS RAPID diffractometer	2711 reflections with *F*^2^ > 2σ(*F*^2^)
ω scans	*R*_int_ = 0.020
Absorption correction: multi-scan (*ABSCOR*; Higashi, 1995)	θ_max_ = 27.5°, θ_min_ = 3.0°
*T*_min_ = 0.785, *T*_max_ = 0.938	*h* = −10→10
9629 measured reflections	*k* = −14→13
2767 independent reflections	*l* = −17→17

### Refinement

**Table:**

Refinement on *F*^2^	Hydrogen site location: inferred from neighbouring sites
*R*[*F*^2^ > 2σ(*F*^2^)] = 0.028	H atoms treated by a mixture of independent and constrained refinement
*wR*(*F*^2^) = 0.074	*w* = 1/[σ^2^(*F*_o_^2^) + (0.045*P*)^2^ + 0.1433*P*] where *P* = (*F*_o_^2^ + 2*F*_c_^2^)/3
*S* = 1.10	(Δ/σ)_max_ < 0.001
2767 reflections	Δρ_max_ = 0.23 e Å^−^^3^
152 parameters	Δρ_min_ = −0.18 e Å^−^^3^
0 restraints	Absolute structure: Flack *x* determined using 1118 quotients [(*I*^+^)-(*I*^-^)]/[(*I*^+^)+(*I*^-^)] (Parsons *et al.*, 2013)
Primary atom site location: structure-invariant direct methods	Absolute structure parameter: 0.01 (9)
Secondary atom site location: difference Fourier map	

### Special details

**Table:**

Geometry. ENTER SPECIAL DETAILS OF THE MOLECULAR GEOMETRY
Refinement. Refinement was performed using all reflections. The weighted *R*-factor (*wR*) and goodness of fit (*S*) are based on *F*^2^. *R*-factor (gt) are based on *F*. The threshold expression of *F*^2^ > 2.0 σ(*F*^2^) is used only for calculating *R*-factor (gt).

### Fractional atomic coordinates and isotropic or equivalent isotropic displacement parameters (Å^2^)

**Table:**

	*x*	*y*	*z*	*U*_iso_*/*U*_eq_	
S1	0.73917 (6)	0.55168 (4)	0.77596 (3)	0.03414 (14)	
O1	0.8795 (2)	0.83178 (13)	0.53057 (12)	0.0499 (4)	
O2	0.9289 (2)	0.65820 (14)	0.45666 (11)	0.0409 (4)	
N1	0.63325 (18)	0.85167 (14)	0.83052 (11)	0.0277 (3)	
N2	0.65342 (19)	0.73327 (14)	0.65780 (10)	0.0261 (3)	
C1	0.5500 (2)	0.74879 (16)	0.82907 (12)	0.0256 (3)	
C2	0.4421 (3)	0.71255 (17)	0.90457 (14)	0.0334 (4)	
C3	0.4220 (3)	0.78569 (19)	0.98617 (14)	0.0363 (4)	
C4	0.5073 (3)	0.89258 (17)	0.98869 (14)	0.0335 (4)	
C5	0.6099 (2)	0.92190 (16)	0.90928 (14)	0.0308 (4)	
C6	0.5841 (2)	0.66856 (16)	0.74195 (12)	0.0263 (3)	
C7	0.7626 (2)	0.65411 (15)	0.60028 (11)	0.0242 (3)	
C8	0.8809 (2)	0.58482 (16)	0.67186 (13)	0.0265 (3)	
C9	0.8626 (2)	0.72556 (17)	0.52484 (12)	0.0295 (4)	
C10	0.9418 (3)	0.46896 (18)	0.62677 (16)	0.0390 (4)	
C11	1.0304 (3)	0.6594 (2)	0.70700 (16)	0.0407 (5)	
H2	0.38344	0.63938	0.90033	0.0401*	
H3	0.35091	0.76278	1.03953	0.0436*	
H4	0.49563	0.94457	1.04353	0.0402*	
H5	0.66695	0.99584	0.91074	0.0369*	
H6	0.47579	0.63008	0.72142	0.0315*	
H7	0.68972	0.59616	0.56409	0.0290*	
H10A	0.84418	0.42248	0.60474	0.0468*	
H10B	1.01526	0.48577	0.57003	0.0468*	
H10C	1.00522	0.42406	0.67652	0.0468*	
H11A	0.98853	0.73348	0.73572	0.0488*	
H11B	1.09424	0.61552	0.75716	0.0488*	
H11C	1.10427	0.67722	0.65067	0.0488*	
H12	0.711 (3)	0.790 (2)	0.6820 (17)	0.0392*	
H13	0.992 (4)	0.696 (3)	0.424 (2)	0.0614*	

### Atomic displacement parameters (Å^2^)

**Table:**

	*U*^11^	*U*^22^	*U*^33^	*U*^12^	*U*^13^	*U*^23^
S1	0.0440 (3)	0.0322 (2)	0.0262 (2)	0.0066 (2)	0.00704 (19)	0.00796 (16)
O1	0.0704 (11)	0.0304 (8)	0.0487 (9)	−0.0018 (7)	0.0192 (8)	0.0082 (7)
O2	0.0512 (9)	0.0432 (8)	0.0282 (7)	−0.0165 (7)	0.0152 (6)	−0.0046 (6)
N1	0.0281 (7)	0.0288 (8)	0.0263 (7)	−0.0001 (6)	0.0014 (6)	0.0008 (6)
N2	0.0305 (7)	0.0296 (8)	0.0183 (6)	0.0036 (6)	−0.0018 (6)	0.0012 (6)
C1	0.0241 (7)	0.0314 (9)	0.0214 (7)	0.0026 (6)	−0.0028 (6)	0.0014 (6)
C2	0.0336 (9)	0.0336 (10)	0.0331 (9)	−0.0037 (8)	0.0061 (8)	0.0012 (8)
C3	0.0391 (10)	0.0418 (11)	0.0281 (9)	0.0034 (9)	0.0105 (8)	0.0032 (8)
C4	0.0402 (10)	0.0344 (10)	0.0259 (8)	0.0106 (8)	0.0019 (7)	−0.0020 (7)
C5	0.0331 (9)	0.0274 (9)	0.0318 (9)	0.0026 (7)	−0.0001 (7)	−0.0002 (7)
C6	0.0262 (8)	0.0310 (8)	0.0216 (8)	−0.0017 (7)	−0.0010 (6)	0.0010 (7)
C7	0.0269 (7)	0.0273 (7)	0.0184 (6)	−0.0040 (7)	−0.0009 (6)	0.0005 (6)
C8	0.0289 (8)	0.0267 (8)	0.0238 (8)	−0.0004 (6)	0.0015 (7)	0.0013 (6)
C9	0.0331 (8)	0.0333 (9)	0.0222 (8)	−0.0035 (7)	−0.0001 (7)	0.0044 (7)
C10	0.0444 (11)	0.0324 (10)	0.0402 (11)	0.0066 (9)	0.0088 (9)	−0.0016 (8)
C11	0.0365 (10)	0.0435 (11)	0.0421 (11)	−0.0056 (9)	−0.0132 (9)	0.0019 (9)

### Geometric parameters (Å, º)

**Table:**

S1—C6	1.8599 (19)	C8—C11	1.528 (3)
S1—C8	1.8362 (19)	O2—H13	0.79 (3)
O1—C9	1.211 (2)	N2—H12	0.85 (2)
O2—C9	1.305 (2)	C2—H2	0.950
N1—C1	1.337 (2)	C3—H3	0.950
N1—C5	1.340 (2)	C4—H4	0.950
N2—C6	1.458 (2)	C5—H5	0.950
N2—C7	1.466 (2)	C6—H6	1.000
C1—C2	1.391 (3)	C7—H7	1.000
C1—C6	1.510 (2)	C10—H10A	0.980
C2—C3	1.387 (3)	C10—H10B	0.980
C3—C4	1.384 (3)	C10—H10C	0.980
C4—C5	1.385 (3)	C11—H11A	0.980
C7—C8	1.556 (2)	C11—H11B	0.980
C7—C9	1.522 (2)	C11—H11C	0.980
C8—C10	1.523 (3)		
			
C6—S1—C8	93.90 (8)	C7—N2—H12	110.5 (16)
C1—N1—C5	117.35 (15)	C1—C2—H2	120.764
C6—N2—C7	109.13 (14)	C3—C2—H2	120.760
N1—C1—C2	123.21 (16)	C2—C3—H3	120.513
N1—C1—C6	116.48 (15)	C4—C3—H3	120.502
C2—C1—C6	120.26 (16)	C3—C4—H4	120.818
C1—C2—C3	118.48 (18)	C5—C4—H4	120.808
C2—C3—C4	118.98 (18)	N1—C5—H5	118.207
C3—C4—C5	118.37 (18)	C4—C5—H5	118.203
N1—C5—C4	123.59 (17)	S1—C6—H6	108.899
S1—C6—N2	107.54 (12)	N2—C6—H6	108.902
S1—C6—C1	110.63 (12)	C1—C6—H6	108.903
N2—C6—C1	111.90 (15)	N2—C7—H7	108.652
N2—C7—C8	109.38 (13)	C8—C7—H7	108.655
N2—C7—C9	109.66 (14)	C9—C7—H7	108.656
C8—C7—C9	111.77 (14)	C8—C10—H10A	109.475
S1—C8—C7	102.22 (12)	C8—C10—H10B	109.471
S1—C8—C10	108.89 (13)	C8—C10—H10C	109.472
S1—C8—C11	110.30 (13)	H10A—C10—H10B	109.472
C7—C8—C10	112.01 (15)	H10A—C10—H10C	109.467
C7—C8—C11	112.32 (15)	H10B—C10—H10C	109.470
C10—C8—C11	110.76 (16)	C8—C11—H11A	109.470
O1—C9—O2	125.42 (18)	C8—C11—H11B	109.475
O1—C9—C7	122.76 (16)	C8—C11—H11C	109.473
O2—C9—C7	111.80 (16)	H11A—C11—H11B	109.465
C9—O2—H13	109 (2)	H11A—C11—H11C	109.474
C6—N2—H12	106.1 (15)	H11B—C11—H11C	109.470

### Hydrogen-bond geometry (Å, º)

*Cg* is the centroid of the N1/C1–C5 ring.

**Table:**

*D*—H···*A*	*D*—H	H···*A*	*D*···*A*	*D*—H···*A*
O2—H13···N2^i^	0.79 (3)	1.87 (3)	2.654 (2)	173 (3)
C3—H3···*Cg*^ii^	0.95	2.81	3.629 (2)	145

Symmetry codes: (i) *x*+1/2, −*y*+3/2, −*z*+1; (ii) *x*−1/2, −*y*+3/2, −*z*+2.
