# Supplementary figures and images for: Crystal structure of (2S,4S)-5,5-dimethyl-2-(pyridin-2-yl)-1,3-thia­zolidine-4-carb­oxy­lic acid
Source: Acta Crystallogr Sect E Struct Rep Online. 2014 Nov 15;70(Pt 12):o1264. doi: 10.1107/S1600536814024854 (PMC4257406; doi:10.1107/S1600536814024854)

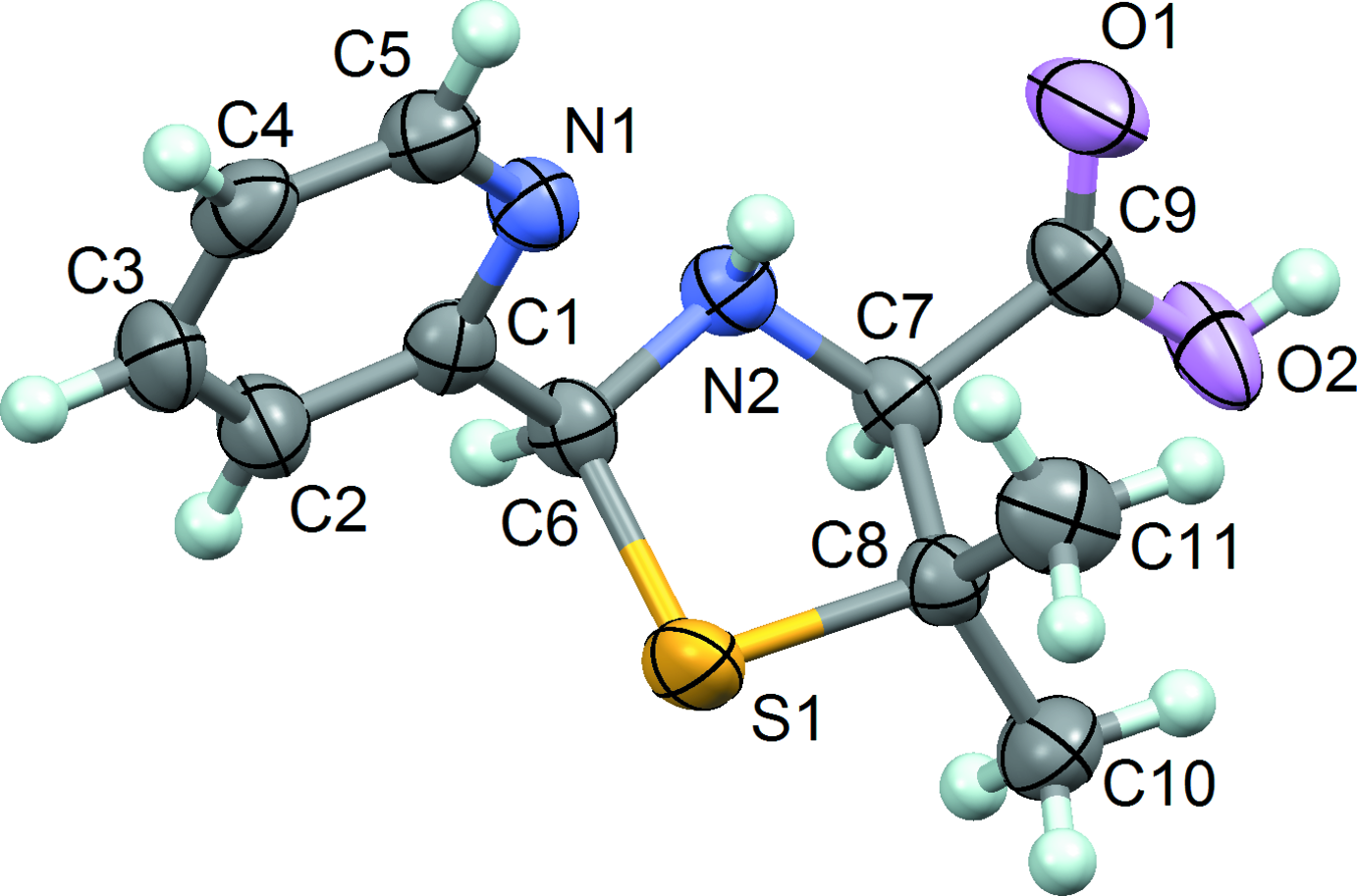

Supplement: Supplementary file 4 [file e-70-o1264-fig1.tif]

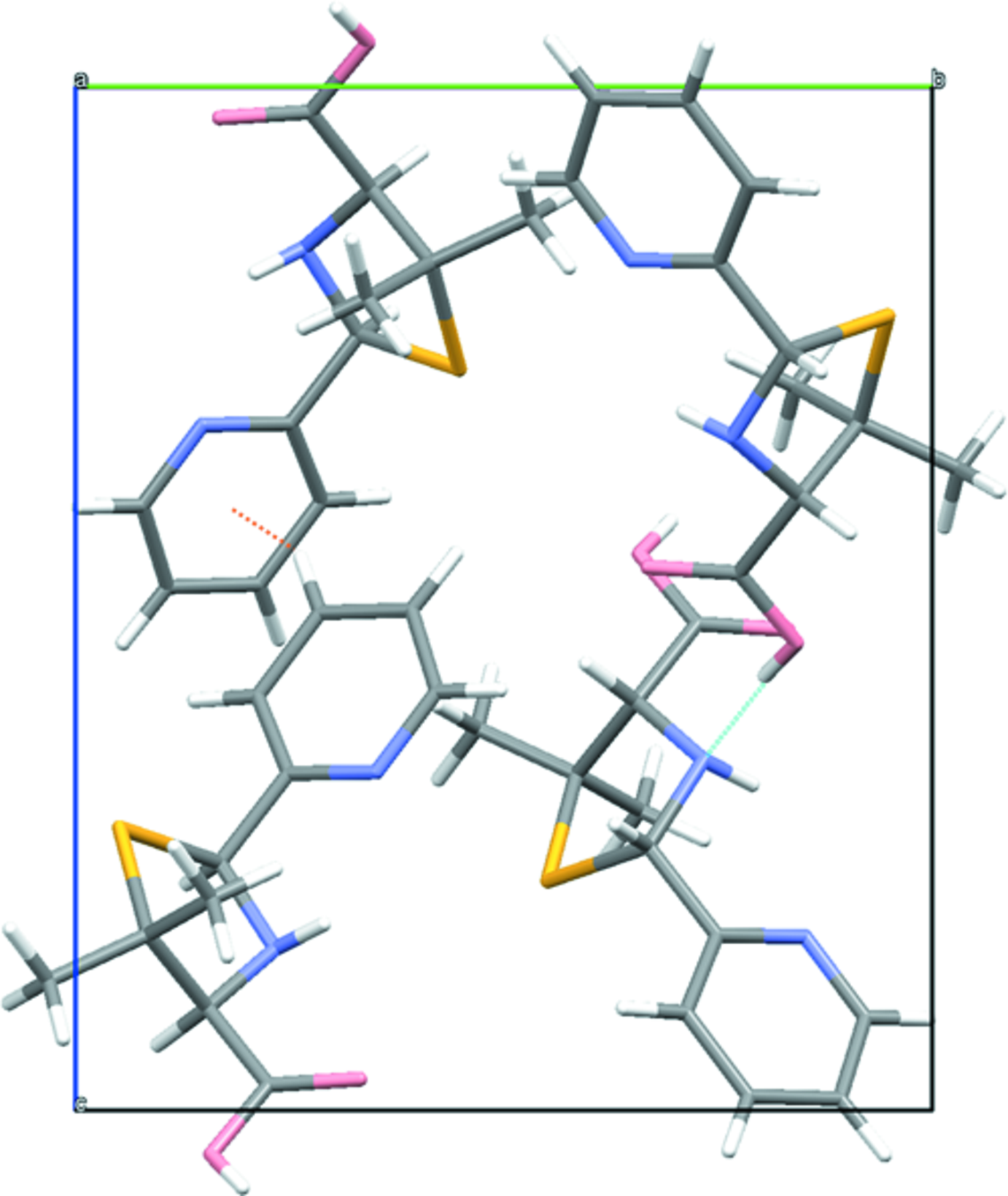

Supplement: Supplementary file 5 [file e-70-o1264-fig2.tif]
